# Supplementary material for: HIV testing uptake and prevalence among hospitalized older adults in Togo: A cross-sectional study
Source: PLoS One. 2021 Feb 2;16(2):e0246151. doi: 10.1371/journal.pone.0246151 (PMC7853528; doi:10.1371/journal.pone.0246151)
Supplement: Attachment — Submitted filename: Revised_Manuscript_With_Track_Changes_PloSOne_OAHIV_ed.pdf [file pone.0246151.s002.pdf]

Full title: HIV testing uptake and prevalence among hospitalized older adults in Togo: a cross-sectional study

Short title: HIV and ageing in Togo

Fifonsi A. GBEASOR-KOMLANVI[1,2], Wendpouire I. C. ZIDA-COMPAORE[2], Arnold J. SADIO[1,2], Martin K. TCHANKONI[2], Balakiyem M. KADANGHA[2], Mounerou SALOU[3], Anoumou C. DAGNRA[3,4], Didier K. EKOUEVI[1,2,5]

1. Université de Lomé, Faculté des Sciences de la Santé, Département de Santé Publique, Lomé, Togo
2. Centre Africain de Recherche en Epidémiologie et en Santé Publique, Lomé, Togo
3. Université de Lomé, Laboratoire de Biologie Moléculaire et d'Immunologie, Lomé, Togo
4. Programme National de Lutte contre le Sida et les Infections Sexuellement Transmissibles, Lomé, Togo
5. INSERM U1219 Bordeaux Population Health Research, ISPED, Université de Bordeaux, Bordeaux, France

Corresponding author

Fifonsi Adjidossi GBEASOR-KOMLANVI, MD, MPH
University of Lomé
Faculty of Health Sciences
Public Health Department
Tel : +228 93 87 41 55
Email : fifonsi.gbeasor@gmail.com

Abstract

Objectives: Data on HIV infection in older adults in West Africa are scarce. This study aimed to describe HIV testing uptake, as well as HIV prevalence and its associated factors among older adults aged ≥50 years in health facilities in Togo.

Methods : A cross-sectional study was carried out from February 2018 to June 2019 among hospitalized older adults aged ≥50 years in tertiary and secondary hospitals in Togo. HIV testing was performed according to the national algorithm. Socio-demographic data and HIV testing history were collected using a standardized questionnaire.

Results: A total of 619 patients (43.9% female) of median age 61 years, (IQR: 55–70) were recruited and offered HIV testing. Among them, 25.7% had never previously tested for HIV. In total, 91.6% (567/619) accepted HIV testing while 8.4% (52/619) refused to be tested. Of those who tested, f~~F~~orty patients were HIV positive, yielding a prevalence of 7.0%, 95%CI: [5.1-9.5]. Twenty-three patients (57.5%) were newly diagnosed with HIV infection. In multivariable analysis, two factors were associated with HIV infection: living alone (aOR=5.83; 95%CI= [2.26-14.53]) and being <60 years (aOR=3.12; 95%CI= [1.51-6.66]).

Conclusion: Nine hospitalized older adults in ten accepted to test for HIV and we observed high HIV prevalence in the study population. There is an urgent need to integrate older adults into responses to the HIV epidemic and to strengthen targeted prevention care and treatment in this population.

Keywords: Ageing; HIV uptake; HIV prevalence; Associated factors; Togo.

Introduction

According to the Joint United Nations Programme on HIV/AIDS (UNAIDS), the number of people living with HIV aged 50 and older (PLWH50+) has increased in all regions of the world [1]. Between 2000 and 2019, the number of PLWH50+ has more than doubled in West and Central Africa, while it has quadrupled in Eastern and Southern Africa; in Western and Central Europe and North America, it showed a ten-fold increase [2]. Globally, the proportion of PLWH50+ increased from 8% of all people living with HIV in 2000 to almost 21% in 2019 [2]. In 2019, there were 7.9 million~~s of~~ PLWH50+ worldwide [2].

The number of older people living with HIV (PLWH) is constantly increasing worldwide, thanks to large-scale access to antiretroviral therapy (ART). Early initiation of ART has led to a decline in AIDS-related deaths by 43% since 2003 and nowadays, ~~a~~ PLWH who starts ART could expect to live as long as an HIV-negative person of the same age [3, 4]. Besides largescale access to ART, ~~with~~ the ageing of the population in all regions of the world~~,~~ has led to new infections ~~are~~ being reported among older adults. In the United States (US), around one in six new HIV diagnoses occurred among older adults in 2017 [5], while an analysis of surveillance data from 31 European countries reported a significant increase in the mean notification rate of new diagnoses among older adults between 2004-2015 [6].

The 'greying of the HIV epidemic' raises important public health issues including ~~.~~ l~~L~~ow rates of HIV testing which have been reported among older adults. In a study conducted among 12,366 people aged ≥50 years in the US, only 25.4% reported lifetime testing for HIV, and of those who had been tested, 69.7% reported having last tested more than five years prior to survey [7]. ~~Among older adults, low rate of HIV testing usually results in a late diagnosis.~~ With regards to new infections, older adults are more likely to be diagnosed at a late stage in the course of the HIV disease than younger people [6, 8]. Late diagnosis among older adults is of great concern as it is associated with adverse outcomes such as rapid progression to AIDS, presence of ageing-related comorbidities and high mortality rate [9–11]. To improve

early detection of HIV infection, especially among populations otherwise unlikely to obtain an HIV test like older adults, implementation of routine HIV testing is the best strategy and it is recommended for everyone between the ages of 13 and 64 in the US [9, 12]. However, less than two older adults in five in the US who tested for HIV were offered HIV testing during routine medical check-up [7].

~~There is a growing research body on HIV and ageing in all regions of the world.~~ In sub-Saharan Africa (SSA) which bears the heaviest burden of the HIV epidemic, evidence on older PLWH is ~~also~~ beginning to emerge [13–20]. Estimates of HIV prevalence among older adults aged ≥50 years have been reported in Tanzania where it ranged from 1.7% [17] to 6.7% [18, 19], in Botswana ~~(~~the rate was 21.9% in females and 27.8% in males [15]) whilst~~,~~ in South Africa it was~~(~~ 20%~~)~~ [20]. ~~Regarding~~ HIV testing among older adults~~, it~~ remains a challenging issue in SSA. Although various HIV testing strategies such as voluntary counselling and testing (VCT), provider-initiated testing and counselling (PITC), diagnostic testing and counselling (DTC), home-based counselling and testing (HBCT), and self-testing are available in SSA countries, HIV testing programs do not specifically target older adults [16, 21, 22]. Also, studies have uncovered that older PLWH in SSA are often unaware of treatment options and availability and they have inadequate access to health services [15]. However, most of these studies were conducted in Eastern and Southern Africa and do not report new HIV infections. In Togo, as in most SSA countries, national adult population surveys on HIV are generally limited to the 15-49 age group and in 2018, the prevalence of HIV was 2.3% [23]. According to the UNAIDS, it was estimated that among the 120,000 PLWH in Togo in 2019, 20,000 (18.3%) were aged ≥50 years [2]. To our knowledge, only one study has focused on the burden of the HIV epidemic among older adults in Togo and reported extrapolated prevalence based on existing data in 2010 [24]. The present study aimed to describe uptake of HIV testing, as well as HIV prevalence and its associated factors among older adults aged ≥50 years in health facilities in Lomé.

Methods

Study design and settings

This cross-sectional study was conducted in Togo, a country of West Africa which covers an area of 56,800 Km² with an average density of 145 inhabitants per square kilometer. The population was 7.89 million in 2018, of which 50.2% are women. Most of the population is young (60% of Togolese are under 25 years of age), and lives in rural areas (62%). Togo health system has a three-level pyramid structure: central, intermediate and peripheral levels. For each level, there are administrative and healthcare delivery components. Regarding health care for older adults, no geriatric wards are available in the country.

This study was part of the Study of Hospitalized Older adults in Togo (SHOT) which aimed to describe comorbidities and mortality after hospitalization among older adults aged ≥50 years in Togo. The SHOT project was carried out in two steps: face-to-face interview at baseline during hospitalization and telephone interviews at one (M1), three months (M3), six months (M6), and twelve months (M12) after hospital discharge. Baseline interviews were conducted from February 2018 to June 2019 in the medicine and surgery hospitalization wards of the three main tertiary level hospitals and secondary level hospitals according to the health pyramid in Togo. Results of HIV testing which was offered at baseline during hospitalization are reported in the present cross-sectional study.

Study population and sample size

Patients aged ≥50 ≥ years who were hospitalized in adult medical and surgical wards of participating hospitals during the study period were eligible to participate in the study. Exclusion criteria were inability to provide consent for HIV testing due to altered mental status or critical medical condition.

Since no data on HIV testing uptake among older adults were available in Togo, the sample size calculation was based on an expected HIV testing uptake in older adults aged ≥50 years

of 73% based on the estimate reported in health facilities in SSA [25], with a precision of 4%, a significance level set at 5%, and a non-response rate of 10%; the minimum sample size was estimated at 522 patients. This sample size was also adequate for the estimation of HIV prevalence among older adults based on the following assumptions: an expected HIV prevalence in older adults aged ≥50 years of 2~~,~~.3% based on the estimate reported in the general population (15-49 years) in Togo [16], with a precision of 2%, a significance level set at 5%, and a non-response rate of 10%, yielding a minimum sample size of 237 patients.

Data collection

Prior to the study, the ten final-year medical students who enrolled the patients received training on HIV testing issues (counselling, obtaining consent, delivering post-test results in coordination with on-site HIV management staff) and on the interview questionnaire. All eligible patients were informed ~~on~~ of the study procedures and were invited to participate ~~in the study.~~ After informed consent ~~approval~~, ~~the medical students recorded~~ sociodemographic data and information regarding HIV testing history, and knowledge of their principal sexual partner's HIV status was recorded. For participants who reported being HIV positive or being tested during their stay at the hospital, medical records were assessed to collect information on their HIV testing history and status.

HIV testing

HIV testing was performed according to the national algorithm in Togo, where~~.~~ ~~In Togo,~~ HIV testing strategies are based on VCT and PICT. PICT is proposed to pregnant women, children aged 18-59 months, key populations and to all other adults. For the present study, HIV testing was proposed to all patients with and without history of HIV testing. ~~Potential study~~ Study participants who refused to be surveyed were asked to provide the reasons ~~of~~ for their refusal.

A small amount of blood was collected by finger prick to perform rapid point-of-care HIV testing using Alere Determine^{TM} HIV1/2 Ag/Ab Combo (Alere Medical Co.Ltd., Chiba, Japan). Each HIV positive test was confirmed with another HIV rapid test, the First

Response® HIV 1-2-O Card Test (Premier Medical Corporation Pvt. Ltd., Maharashtra, India). In case of discordant results, venous blood samples were drawn and tested with the INNO-LIA® HIV I/II Score (20T) (Fujirebio, Göteborg, Sweden) line immunoassay at the main HIV laboratory research unit, the Molecular Biology Laboratory (BIOLIM) at the University of Lomé.

Counselling and risk prevention interventions were provided to patients who were tested negative for HIV. Positive results were disclosed by a senior medical officer in charge in the service where newly HIV positive patients were diagnosed. Senior medical officers also emphasized the benefits of medical care and treatment before referring newly diagnosed HIV patients to the HIV care center.

Statistical analysis

Descriptive statistics were performed and results were presented with frequency tabulations and percentages for categorical variables. Quantitative variables were presented as medians with their interquartile range (IQR). HIV prevalence was estimated with corresponding 95% confidence interval (95%CI). Comparisons of medians were performed using Wilcoxon-Mann-Whitney test and proportions were compared using Chi-square test or Fisher's exact test. Binary logistic regression analyses were performed to identify factors associated with HIV infection among older adults. In the univariable logistic regression, variables with a *p*-value < 0.20 were fitted into the multivariable analyses. A backward procedure approach was performed for selection of variables and adjusted odds ratio (aOR) were reported with their 95% CI. All analyses were performed using R® software. The significance level was set at 5%.

Ethical considerations

All eligible patients received detailed information about the study purpose and procedures, voluntary participation, HIV testing, potential risks and protections. Recruited patients provided written signed informed consent prior to the administration of the questionnaire and

additional consent was obtained prior to HIV testing. This study was approved by the "Comité de Bioéthique pour la Recherche en Santé (CBRS)" (Bioethics Committee for Health Research) from the Togo Ministry of Health (n°09/2018/CBRS).

Results

A total of 659 eligible older adults were recruited for the purpose of the SHOT project during the study period. Forty older adults were unable to provide consent for HIV testing because of their general health status (coma, stroke) and a total of 619 adults were offered HIV testing and included in the analysis.

Socio-demographic characteristics

The median age of the 619 ~~selected older adults~~participants was 61 years, (IQR: 55–70). Women represented 43.9% of the sample and they were more likely to be widowed~~,~~ and have no formal or primary level education ($p<0.001$). Almost three quarters (73.8%) of the study population did not have health insurance (Table 1).

Table 1. Socio-demographic characteristics of hospitalized older adults in Lomé, Togo in 2018-2019 (N=619)

	Total (N=619)		Male (N=347)		Female (N=272)		P-value
	N	%	N	%	N	%	
**Age (years), median (IQR)**	61 (55-70)		60 (55-68)		62 (57-70)		0.002*
**Age (years)**							0.021**
[50-60[	262	42.3	161	46.4	101	37.1	
≥ 60	357	57.7	186	53.6	171	62.9	
**City of residence**							0.513**
Lomé	359	58.0	204	58.8	155	57.0	
Other cities	182	29.4	104	30.0	78	28.7	
MD	78	12.6	36	11.2	39	14.3	
**Marital status**							<0.001***
Married/Living with a partner	403	65.1	304	87.6	99	36.4	
Widow	170	27.5	27	7.8	143	52.6	
Living alone	43	6.9	14	4.0	29	10.7	
MD	3	0.5	2	0.6	1	0.3	
**Education level**							<0.001**
No formal education	173	27.9	48	13.8	125	46.0	
Primary school	158	25.5	88	25.4	70	25.7	
Secondary/ University	275	44.4	204	58.8	71	26.1	
MD	13	2.2	7	2.0	6	2.2	
**Main source of monthly revenue**							<0.001**
None	31	5.0	11	3.2	20	7.4	
Financial aid	184	29.7	71	20.5	113	41.5	
Pension fund	103	16.6	83	23.9	20	7.4	
Income generating activity	278	45.0	169	48.7	109	40.1	
MD	23	3.7	13	3.7	10	3.6	
**Monthly income (Euros)**							<0.001**
< 53	136	22.0	58	16.7	78	28.7	
[53-153[	329	53.2	189	54.5	140	51.5	
≥> 153	118	19.1	84	24.2	34	12.5	
MD	36	5.7	16	4.6	20	7.3	
**Health insurance**							<0.001***
Yes	157	25.4	120	34.6	37	13.6	
No	457	73.8	225	64.8	232	85.3	
MD	5	0.8	2	0.6	3	1.1	

*Mood test; **Chi-square test; ***Fisher test; MD: missing data

History of HIV testing and uptake

Among the 619 older adults who were included in the study, 159 (25.7%) had never been tested for HIV. In total, 91.6% (567/619) accepted HIV testing while 8.4% (52/619) refused to be tested; there was a statistical difference of HIV refusal proportions (p=0.018) according to HIV history. Main reasons for HIV test refusal were ‘I do not want to be tested again’, ‘I am not at risk’, or there was no specific reason. History of HIV testing and uptake are summarized in Figure 1.

HIV prevalence

In total, 40 hospitalized older adults (of~~ver~~ 567 tested) were ~~infected with~~positive for HIV, yielding a prevalence of 7.0%, 95%CI: [5.1-9.5]. Among HIV-~~infected~~ seropositive older adults, 17 were known HIV positive.

HIV prevalence was higher among older adults who were living alone (22.2%) compared with those who were widowed (5.7%) or married/living with a partner (5.9%) (p<0.001). Also, HIV prevalence was higher in those aged 50-59 years compared with their older counterparts (respectively 11.0% and 4.2%; p=0.002). There was no statistical difference according to sex (6.9% in male, 7.2% in female; p=0.954).

~~***Factors associated with HIV prevalence***~~

In multivariable analysis, two factors were associated with HIV infection: marital status (living alone) (aOR=5.83; 95%CI= [2.26-14.53]) and being <60 years of age (aOR=3.12; 95%CI = [1.51-6.66]). These results are presented in the T~~t~~able 2.

Table 2. Factors associated with HIV prevalence among hospitalized older adults in Lomé, Togo in 2018-2019

	HIV prevalence (%) N=567	Univariable analysis			Multivariable (full model) analysis			Multivariable (final model) analysis		
		OR	95%CI	p-value	aOR	95%CI	P-value	aOR	95%CI	p-value
**Age (years)**										
≥ 60	4.2	1			1			1		
[50-60[	11.0	3.12	[1.58-6.25]	0.001	3.33	[1.58-7,14]	0.002	3.12	[1.51-6.66]	0.002
**Sex**										
Male	6.9	1			1			1		
Female	7.2	1.07	[0.56-2.05]	0.832	0.65	[0.27-1.50]	0.318	0.72	[0.32-1.59]	0.418
**Wards**										
Medicine	8.0	1			1			1		
Surgery	3.7	0.39	[0.11-1.0]	0.079	0.37	[0.11-0.99]	0.074	0.38	[0.11-0.99]	0.075
**Education level**										
No formal education	7.3	1			1			-		
Primary school	6.4	0.84	[0.34-2.05]	0.710	0.66	[0.25-1.70]	0.391	-	-	-
Secondary/University	7.6	1.03	[0.49-2.24]	0.944	0.71	[0.30-1.73]	0.442	-	-	-
**Marital status**										
Married/Living with a partner	5.9	1			1			1		
Widowed	5.7	1.23	[0.54-2.65]	0.604	1.75	[0.66-4.52]	0.252	1.79	[0.68-4.60]	0.231
Living alone	22.2	6.23	[2.61-14.21]	<0.001	5.92	[2.29-14.87]	<0.001	5.83	[2.26-14.53]	<0.001

95%CI: 95% confidence interval; OR: Odds Radio; aOR: adjusted Odds Ratio

Discussion

The present study explored uptake of HIV testing and the prevalence of HIV infection among hospitalized older adults in tertiary and secondary hospitals in Togo in 2018-2019. More than 9.2% of hospitalized patients refused HIV testing and HIV prevalence was estimated at 7.0%, which was more than three times the HIV prevalence in the general population (2.3% in 2018) among people aged 15-49 years. Factors associated with positive HIV status were living alone and being aged <60 years old.

Reaching the first 90 – 90% of people living with HIV know their status – remains a challenge in SSA, especially for older adults. Data in West and Central Africa in 2019 estimated that 68% of people living with HIV were aware of their status [26]. ~~In older adults, o~~One in four hospitalized older adults (25.7%) in our study had never been tested for HIV. This could be explained by the fact that HIV testing was not offered systematically to older adults. A study conducted in South Africa included 4,560 participants; the combined cascade indicated a high HIV testing rate (89% (95% CI 87 to 91), but lower knowledge of HIV-positive status (71% (95% CI 68 to 74) [27]. A qualitative study in Western Kenya of 57 HIV positive people aged ≥50 years ~~. This study~~ reported that experience ~~in~~ of HIV testing ~~on~~ in older adults depended on where they tested (hospital or community setting), whether they actively sought the testing or not,~~;~~ and on the age and gender of the healthcare provider who conducted the test [22]. ~~PITC should also focus on older adults whatever the age and the marital status.~~ ~~Indeed,~~ HIV testing and prevention initiatives have historically focused on younger population and key populations PITC should also ~~focus on~~include older adults ~~whatever the age and~~irrespective of their marital status.~~.~~

Refusal of HIV testing also constitutes a challenging issue. In Botswana, among the barriers to HIV testing, 76.8% felt that they were not at risk of HIV infection or did not have time to go to the testing center (6.5%) [28]. In Zambia, 31.6% of the 44,791 participants in the tuberculosis demographic survey refused HIV testing. Qualitative research is needed to better understand the reasons for refusal and to identify specific interventions to increase acceptance

of HIV testing. In general, acceptance of HIV testing is influenced by individuals' attitudes regarding confidential concerns, social support, and stigma associated with HIV testing [29]. More than two third of HIV testing refusals were reported in an emergency department in the Eastern Cape in South Africa; this clearly shows the challenge of PICT [30]. ~~It could be explained by the fact that~~ O~~o~~lder adults may not consider themselves at risk or may mistake HIV symptoms for those of normal ageing and not consider HIV as a potential cause [31]. In western Kenya, older adults expressed concerns with ageist discrimination specifically in hospital settings, characterized by providers' reluctance or refusal to test [22]. Thus, HIV campaign messages do not target older adults and may not have the same impact on older populations.

In the US, based on the 2009 National Health Interview Survey, only 52.7% of HIV tests performed during a routine medical check-up were suggested by a health care provider [7]. Such missed opportunities represent a very concerning issue considering the increasing number of older PLWH and HIV prevalence among people aged ≥50 years [14, 15, 32, 33]. ~~In Canada, for example, between 2008 and 2017, new HIV diagnoses have increased among males and females aged ≥50 years, while decreasing among younger adults over the same time period [27].~~ Like younger people, older adults also need HIV services, but their needs are often overlooked, neglected or ignored [34]. In fact, concerning older adults, much of the focus is on non-communicable diseases than on HIV infection. Although sexual transmission has been reported as the main route of transmission among older adults [6], they are often perceived as asexual patients by health care providers and are less likely to be offered HIV testing [35, 36]. Also, health care providers feel uncomfortable discussing sexual history, especially with older adults [37]. The low HIV testing demonstrates that PITC approach as recommended by WHO does not target older adults in Togo [38]. There is a need to reinforce universal offering of HIV testing among hospitalized patients in order to detect undiagnosed

cases at an early stage. Also, an alternative approach with HIV self-testing could be proposed [39].

There are limited data on HIV infection in older adults in Africa as those aged ≥50 years have been largely excluded from epidemiological studies, especially in West Africa. In a rural region of Cameroon, a prevalence of 2.6% was reported among people aged 55 years and over [40]. Another cross-sectional study which used data from the baseline measurement of the Ifakara MZIMA cohort study in 2012/13 in rural area in Tanzania and included 1,643 adults 50+ years, reported HIV prevalence and testing rate (ever tested) of 6% and 11.4% respectively [41]. In Zimbabwe, HIV prevalence was high among men aged 45-54 years (23.4 percent) compared to those aged 15-44 years (11.0 percent) [14]. A similar observation was made among women aged 45-54 years (21.3%) between 2009 and 2011, compared to women aged 15-44 years (17.3%). In addition, this same study reported HIV incidence among men aged ≥45 years and older was 1.03 per 1,000 person-years, which was non-significantly (p>0.05) lower than among younger men aged 15-44 years (1.27 person-years). Hence, with a prevalence among 15 to 49 years old of 2.3% in Togo and the prevalence of 7% found on this study, older people should be included in HIV prevention and treatment programs. As reported in Zimbabwe, this high prevalence could be explained by a range of behaviors such as low condom use and a relatively higher rate of multiple partnerships that put older adults at risk of HIV infection [14]. A study conducted in Kenya, based on the government's 2012 HIV update report, indicated that 5,000 to 15,000 older people were newly infected in 2011 out of a total of 91,000 new infections and therefore estimated that 55% to 16.5% of new infections are among people aged 50 years and older [42]. In Botswana, HIV prevalence among adults aged 50-64 years was 24.6 percent in 2013, compared with 35.1 percent among young adults (25-49 years). Prevalence was higher among older men (27.8%) than among women (21.9%), p=0.002. In this study, 58.9% of older adults admitted to being sexually active, with 59.0% admitting to inconsistent condom use during sexual intercourses [15]. A recent review of data from 40 Demographic and Health Surveys (DHS) conducted in 27 sub-

Saharan African countries between 2003 and 2012 found that HIV prevalence among adults (45-59 years) was higher than in the general adult population, except in Democratic Republic of Congo, Ethiopia, Mozambique, Sierra Leone, and Swaziland [32]. We reported HIV prevalence in hospitals, which is generally higher than that of the general population. Surveys including older adults have found HIV prevalence ranging from 5% among 50-64 years old in Kenya to 13% among 50-54 years old in South Africa. In Swaziland, prevalence was 6.4% among adults aged 50 years or older, 13% among men, and 7% among women aged 60-64 years [43].

In South Africa, a secondary data analysis based on the 2012 population-based nationally representative multi-stage stratified cluster random household sample showed that increased risk of HIV was significantly associated with age group 25-49 years and those 50 years and older compared with young males 15-25 years [44]. In SSA, data from Demographic and Health Surveys (DHS) reported a prevalence of 4.0% among people aged >50 years [24]. In the present study, one hospitalized older adult in 20 was infected with HIV and similarly, a study conducted in a hospital in Dares-Salam in Tanzania reported high prevalence of 15% among those aged 55 years and older [45]. However, comparisons should be made with caution since the recruitment settings or strategies differ from one country to another. Nevertheless, these results showed the need to focus prevention strategy in older adults.

Few studies have reported data on HIV prevalence stratified by age among older adults in SSA. Globally, the prevalence of HIV decreases with age after the age of 50 years based on available data in African countries [24]. In 2008, the prevalence of HIV was 10.4% in male aged 50-54 years, 6.2% in male aged 55-59 years, and 3.5% in male aged ≥60 years in South Africa [24]. Similar trends were reported in Kenya, Rwanda, and in Uganda [24]. However, HIV prevalence is increasing over the years in older age groups in SSA [15, 24]. Intergenerational sex or having sex with partners at least 10 years younger is a risky and frequent sexual behavior among older adults [13, 46], which could explain this high

prevalence. Also, this increased prevalence may be related to practices such as wife inheritance, which are common in SSA and where a widow marries a deceased's relative [13]. In the present study, older adults who were living alone had six times the odds of being infected with HIV compared to their counterparts who were married or living with a partner. Similarly in South Africa, it was reported that married individuals who were living with their spouse had significantly reduced odds of being HIV positive compared to all other marital status groups [47]. This could be explained by the fact that single individuals are more likely to have high numbers of sexual partners and that older adults inconsistently use condom during sexual intercourse [15, 34].

This study has some limitations. First, we used the national algorithm for HIV diagnosis, which does not allow to identify recent HIV infection [48]. Second, we recruited older adults in clinical settings; therefore, HIV prevalence estimates could not be generalized to all older adults in Togo and could be overestimated. However, this study documented missed opportunities of HIV testing among hospitalized older adults. Also, it provided an overview of HIV prevalence in hospitals in Togo and the need to test all older adults.

Conclusion and recommendations

This study explored HIV testing uptake, HIV prevalence and its associated factors among older adults in hospitals in Togo. We observed high prevalence of HIV contrasting with low uptake of HIV testing among the study population. Achieving the 90:90:90 targets requires identifying the largest possible number of HIV-positive individuals, including older adults. ~~The delivery of services to older adults with HIV infection need to be improved in order to avoid stigma and initiate appropriate care.~~ Also, management of HIV infection among older adults deserves great attention as for non-communicable diseases. Thus, there is an urgent need to integrate older adults into responses to the HIV epidemic and to strengthen targeted prevention care and treatment in this population. Futures studies should better document virological responses in older adults in Africa since immunological responses seems to be

influenced by older age [49]. In addition, the relationship between HIV infection and sexual practices among older adults should be more documented especially in West Africa.

Abbreviations

95% CI: 95% confidence interval

aOR: adjusted Odds Ratio

IQR: interquartile range

MD: missing data

PICT: provider-initiated testing and counselling

PLWH: people living with HIV

PLWH50+: people living with HIV aged 50 years and older

SSA: sub-Saharan Africa

VCT: voluntary counselling and testing

Supporting information

S1 Dataset. HIV testing uptake and prevalence among hospitalized older adults in Togo: a cross-sectional study.

Acknowledgments

We are thankful to the older adults who accepted to participate in this study. We are also thankful to the medical students of the 'Faculté des Sciences de la Santé-Université de Lomé' who performed data collection for the study.

We are deeply indebted to the "Centre Africain de Recherche en Epidémiologie et en Santé Publique" (CARESP) and the "Programme National de Lutte contre le sida, les hépatites virales et les infections sexuellement transmissibles du Togo" (PNLS/HV/IST) who provided technical and logistical support to this work.

Conflicts of interest

The authors declare they have no competing interests.

REFERENCES

1. Joint United Nations Programme on HIV/AIDS. HIV and aging [Internet]. 2013 [Accessed 19 Feb 2020]. Available from: http://www.unaids.org/en/media/unaids/contentassets/documents/unaidspublication/2013/20131101_JC2563_hiv-and-aging_en.pdf.

2. Autenrieth CS, Beck EJ, Stelzle D, Mallouris C, Mahy M, Ghys P. Global and regional trends of people living with HIV aged 50 and over: Estimates and projections for 2000–2020. PLOS ONE. 2018;13:e0207005.

3. Joint United Nations Programme on HIV/AIDS. Global AIDS Update [Internet]. 2016 [Accessed 19 Feb 2020]. Available from: https://www.unaids.org/sites/default/files/media_asset/global-AIDS-update-2016_en.pdf.

4. Nakagawa F, May M, Phillips A. Life expectancy living with HIV: recent estimates and future implications. Curr Opin Infect Dis. 2013;26:17–25.

5. Center for Disease Control and Prevention. HIV Among People Aged 50 and Over [Internet]. 2018 [Accessed 19 Feb 2020]. Available from: https://www.cdc.gov/hiv/group/age/olderamericans/index.html.

6. Tavoschi L, Dias JG, Pharris A, Schmid D, Sasse A, Beckhoven DV, et al. New HIV diagnoses among adults aged 50 years or older in 31 European countries, 2004–15: an analysis of surveillance data. Lancet HIV. 2017;4:e514–21.

7. Adekeye OA, Heiman HJ, Onyeabor OS, Hyacinth HI. The New Invincibles: HIV Screening among Older Adults in the U.S. PLOS ONE. 2012;7:e43618.

8. Hu X, Liang B, Zhou C, Jiang J, Huang J, Ning C, et al. HIV late presentation and advanced HIV disease among patients with newly diagnosed HIV/AIDS in Southwestern China: a large-scale cross-sectional study. AIDS Res Ther. 2019;16:6.

9. Ford CL, Godette DC, Mulatu MS, Gaines TL. Recent HIV Testing Prevalence, Determinants, and Disparities Among U.S. Older Adult Respondents to the Behavioral Risk Factor Surveillance System. Sex Transm Dis. 2015;42:405–10.

10. Metallidis S, Tsachouridou O, Skoura L, Zebekakis P, Chrysanthidis T, Pilalas D, et al. Older HIV-infected patients—an underestimated population in northern Greece: epidemiology, risk of disease progression and death. Int J Infect Dis. 2013;17:e883–91.

11. Brooks JT, Buchacz K, Gebo KA, Mermin J. HIV Infection and Older Americans: The Public Health Perspective. Am J Public Health. 2012;102:1516–26.

12. Center for Disease Control and Prevention. HIV Testing [Internet]. 2020 [Accessed 19 Feb 2020]. Available from: https://www.cdc.gov/hiv/testing/index.html.

13. Mutevedzi PC, Newell M-L. A missing piece in the puzzle: HIV in mature adults in sub-Saharan Africa. Future Virol. 2011;6:755–67.

14. Negin J, Gregson S, Eaton JW, Schur N, Takaruza A, Mason P, et al. Rising Levels of

HIV Infection in Older Adults in Eastern Zimbabwe. PLoS ONE. 2016;11. doi:10.1371/journal.pone.0162967.

15. Matlho K, Randell M, Lebelonyane R, Kefas J, Driscoll T, Negin J. HIV prevalence and related behaviours of older people in Botswana - secondary analysis of the Botswana AIDS Impact Survey (BAIS) IV. Afr J AIDS Res AJAR. 2019;18:18–26.

16. Wandera SO, Kwagala B, Maniragaba F. Prevalence and determinants of recent HIV testing among older persons in rural Uganda: a cross-sectional study. BMC Public Health. 2020 1;20(1):144. doi:10.1186/s12889-020-8193-z.

17. Swai SJ, Damian DJ, Urassa S, Temba B, Mahande MJ, Philemon RN, et al. Prevalence and risk factors for HIV among people aged 50 years and older in Rombo district, Northern Tanzania. Tanzania Journal of Health Research. 2017;19(2). doi.org/10.4314/thrb.v19i2.

18. Mtowa A, Gerritsen AA, Mtenga S, Mwangome M, Geubbels E. Socio-demographic inequalities in HIV testing behaviour and HIV prevalence among older adults in rural Tanzania, 2013. AIDS care. 2017;2;29(9):1162-8.

19. Mtenga SM, Pfeiffer C, Merten S, et al. Prevalence and social drivers of HIV among married and cohabitating heterosexual adults in south-eastern Tanzania: analysis of adult health community cohort data. Glob Health Action. 2015;8:28941. doi:10.3402/gha.v8.28941

20. Rosenberg MS, Gómez-Olivé FX, Rohr JK, Houle BC, Kabudula CW, Wagner RG, et al. Sexual behaviors and HIV status: a population-based study among older adults in rural South Africa. Journal of acquired immune deficiency syndromes (1999). 2017 1;74(1):e9-e17. doi:10.1097/QAI.0000000000001173

21. Nabukenya AM, Matovu JKB. Correlates of HIV status awareness among older adults in Uganda: results from a nationally representative survey. BMC Public Health. 2018;18(1):1128. doi:10.1186/s12889-018-6027-z

22. Kiplagat J, Huschke S. HIV testing and counselling experiences: a qualitative study of older adults living with HIV in western Kenya. BMC Geriatr. 2018;18(1):257. doi:10.1186/s12877-018-0941-x

23. Joint United Nations Programme on HIV/AIDS. Togo [Internet]. 2020 [Accessed 19 Feb 2020]. Available from: https://www.unaids.org/fr/regionscountries/countries/togo.

24. Negin J, Cumming RG. HIV infection in older adults in sub-Saharan Africa: extrapolating prevalence from existing data. Bull World Health Organ. 2010;88:847–53.

25. Sharma M, Ying R, Tarr G, Barnabas R. Systematic review and meta-analysis of community and facility-based HIV testing to address linkage to care gaps in sub-Saharan Africa. Nature. 2015;528:S77-85.

26. AVERT. HIV and AIDS in West and Central Africa overview. [Internet]. 2020 [Accessed 16 Sep 2020]. Available from: https://www.avert.org/hiv-and-aids-west-and-central-africa-overview.

27. Rohr JK, Manne-Goehler J, Gómez-Olivé FX, Wagner RG, Rosenberg M, Geldsetzer P, et al. HIV treatment cascade for older adults in rural South Africa. Sex Transm Infect. 2020;96(4):271-276.

28. Ama, Njoku & Shaibu, Sheila. Knowledge and utilization of voluntary counselling and testing services for HIV by older adults (50 Years and Over) in Botswana. Journal of AIDS & Clinical Research. 2015; 6(10): 511. doi: 10.4172/2155-6113.1000511.

29. Chanda-Kapata P, Ngosa W, Ngomah Moraes A, Maddox N, and Kapata N. Decliners of provider-initiated HIV testing and counselling: Characteristics of participants who refused HIV testing in a population survey in Zambia. Asian Pacific Journal of Tropical Biomedicine. 2015;5(8):689-693.

30. Ryan S, Hahn E, Rao A, Mwinnaa G, Black J, Maharaj R, et al. The impact of HIV knowledge and attitudes on HIV testing acceptance among patients in an emergency department in the Eastern Cape, South Africa. BMC Public Health. 2020;20(1):1066. doi:10.1186/s12889-020-09170-x.

31. Center for Disease Control and Prevention. HIV and older Americans. [Internet]. 2020 [Accessed 16 Sept 2020]. Available from: https://www.cdc.gov/hiv/group/age/olderamericans/index.html

32. Vollmer S, Harttgen K, Alfven T, Padayachy J, Ghys P, Bärnighausen T. The HIV Epidemic in Sub-Saharan Africa is Aging: Evidence from the Demographic and Health Surveys in Sub-Saharan Africa. AIDS Behav. 2017;21 Suppl 1:101–13.

33. Mahy M, Autenrieth CS, Stanecki K, Wynd S. Increasing trends in HIV prevalence among people aged 50 years and older: evidence from estimates and survey data. AIDS Lond Engl. 2014;28 Suppl 4:S453-459.

34. Joint United Nations Programme on HIV/AIDS. The Gap Report: People aged 50 years and older [Internet]. 2014 [Accessed 19 Feb 2020]. Available from: https://www.unaids.org/sites/default/files/media_asset/12_Peopleaged50yearsandolder.pdf.

35. Youssef E, Wright J, Delpech V, Davies K, Brown A, Cooper V, et al. Factors associated with testing for HIV in people aged ≥50 years: a qualitative study. BMC Public Health. 2018;18. doi:10.1186/s12889-018-6118-x.

36. Akanji BO, Ogunniyi A, Baiyewu O. Healthcare for older persons, a country profile: Nigeria. J Am Geriatr Soc. 2002;50:1289–92.

37. Gott M, Hinchliff S, Galena E. General practitioner attitudes to discussing sexual health issues with older people. Soc Sci Med 1982. 2004;58:2093–103.

38. World Health Organization. Guidance on provider-initiated HIV testing and counselling in health facilities [Internet]. 2007 [Accessed 19 Feb 2020]. Available from: https://apps.who.int/iris/bitstream/handle/10665/43688/9789241595568_eng.pdf;jsessionid=A416AB893CCF6236F0BE94A75835467F?sequence=1.

39. Ekouevi DK, Bitty-Anderson A, Gbeasor-Komlanvi FA, Coffie AP, Eholie SP. HIV self-testing: the key to unlock the first 90 in West and Central Africa. Int J Infect Dis.

2020;0. doi:10.1016/j.ijid.2020.02.016.

40. Nyambi P, Zekeng L, Kenfack H, Tongo M, Nanfack A, Nkombe I, et al. HIV infection in rural villages of Cameroon. J Acquir Immune Defic Syndr 1999. 2002;31:506–13.

41. Mtowa A, Gerritsen AAM, Mtenga S, Mwangome M, Geubbels E. Socio-demographic inequalities in HIV testing behaviour and HIV prevalence among older adults in rural Tanzania, 2013. AIDS Care. 2017;29:1162–8.

42. National Aids Control Council (NACC) and National AIDS and STI Control Programme (NASCOP). The Kenya AIDS epidemic update 2012. [Internet]. 2012 [Accessed 16 Sep 2020]. Available from: http://guidelines.health.go.ke:8000/media/FINAL_Kenya_Update_2012_30_May.pdf

43. Harris TG, Rabkin M, El-Sadr WM. Achieving the fourth 90: healthy aging for people living with HIV. AIDS. 2018;32(12):1563-1569.

44. Mabaso M, Makola L, Naidoo I, Mlangeni LL, Jooste S, Simbayi L. HIV prevalence in South Africa through gender and racial lenses: results from the 2012 population-based national household survey. Int J Equity Health. 2019;18:167.

45. Mtei LN, Pallangyo KP. HIV infection in elderly medical patients. East Afr Med J. 2001;78:144–7.

46. Schaefer R, Gregson S, Eaton JW, Mugurungi O, Rhead R, Takaruza A, et al. Age-disparate relationships and HIV incidence in adolescent girls and young women: evidence from Zimbabwe. AIDS Lond Engl. 2017;31:1461–70.

47. Shisana O, Risher K, Celentano DD, Zungu N, Rehle T, Ngcaweni B, et al. Does marital status matter in an HIV hyperendemic country? Findings from the 2012 South African National HIV Prevalence, Incidence and Behaviour Survey. AIDS Care. 2016;28:234–41.

48. World Health Organization. When and how to use assays for recent infection to estimate HIV incidence at a population level [Internet]. 2011 [Accessed 19 Feb 2020]. Available from: https://www.who.int/diagnostics_laboratory/hiv_incidence_may13_final.pdf.

49. Balestre E, Eholié SP, Lokossue A, Sow PS, Charurat M, Minga A, et al. Effect of age on immunological response in the first year of antiretroviral therapy in HIV-1-infected adults in West Africa. AIDS Lond Engl. 2012;26:951–7.
