# Supplementary material for: HIV testing uptake and prevalence among hospitalized older adults in Togo: A cross-sectional study
Source: PLoS One. 2021 Feb 2;16(2):e0246151. doi: 10.1371/journal.pone.0246151 (PMC7853528; doi:10.1371/journal.pone.0246151)
Supplement: Attachment — Submitted filename: Editor_comments_OAVIH.docx [file pone.0246151.s003.docx]

**Additional Editor Comments**

*The manuscript has been substantially improved with the changes made in response to the previous Reviewers' suggestions. The scientific validity of the data presented is sound, and the findings of interest. However, sections of the manuscript, particularly the Discussion, still require significant changes if the article is to be published. I have addressed some specific instances of phrasing and grammar that require attention as edits in the attached document, but also make some additional suggestions below to try and restructure the Discussion.*

**Authors: We thank the editor for these encouraging comments.**

Lines 47-49: See text edits.

Line 70: See text edit.

Line 262: Remove sentence “refusal of testing…”
Line 264: Study in Zambia not relevant to testing in older people – remove.
Line 269-70: Can remove sentence.

Line 248: Remove sentence beginning “Data in…”

Line 362: The statement that HIV testing uptake was low doesn’t seem justified – remove.

Line 289: Can remove sentence beginning “The low testing…”

Lines 277-284: Could be removed.

Line 252-4/Ref 27: If these data are not specific to older people, please remove.

**Authors: We thank the editor for text edits. We have edited and removed the text accordingly.**

Line 49: 40/567 is 7.1% - please correct. Also, how is there a confidence interval for this result.

**Authors: We thank the editor for this comment. In many studies, we use a sample to estimate population parameters. Any measurement taken from the sample group therefore provides an imprecise estimate of the true population value. We provide references for the rationale for confidence interval estimation for a prevalence.**

- Hazra A. Using the confidence interval confidently. *J Thorac Dis*. 2017;9(10):4125-4130. doi:10.21037/jtd.2017.09.14
- Department of Statisitics. Penn State Eberly College of Science. Introduction to confidence intervals [Internet]. 2020 [Accessed 30 Nov 2020]. Available from: https://online.stat.psu.edu/stat200/lesson/4/4.2
- Eayres D. Technical briefing 3 : Commonly used public health statistics and their confidence intervals [Internet]. 2008 [Accessed 30 Nov 2020]. Available from: http://www.ukiacr.org/publication/technical-briefing-3-commonly-used-public-health-statistics-and-their-confidence

Line 53: The revised sentence should probably be reworded to eg “The majority of older adults in this study accepted testing for HIV and 4.1% of those tested were newly diagnosed with HIV as a result of this test” or similar.

**Authors: We thank the editor for corrections and suggestions. We have corrected the data and we have reworded the sentence in the conclusion of the abstract.**

Of those who tested, forty patients were HIV positive, yielding a prevalence of 7.1%, 95%CI: [5.1-9.5].

The majority of older adults in this study accepted testing for HIV and almost three in five HIV positive older adults were newly diagnosed infected with HIV.

Line 72: This line is now repetitive of the sentences above. Please rephrase to state ART has contributed to the abovementioned increases in numbers of PLWH 50+.

Largescale access to antiretroviral therapy (ART) has greatly contributed to increases in the number of older people living with HIV (PLWH).

Line 78: State the age definition of older adults in Reference 5.

In the United States (US), around one in six new HIV diagnoses occurred among people aged ≥50 years in 2017 [5].

Line 103: Here and elsewhere remove abbreviations where they are used only once.

Although various HIV testing strategies such as voluntary counselling and testing (VCT), provider-initiated testing and counselling (PITC), diagnostic testing and counselling, home-based counselling and testing, and self-testing are available in SSA countries, HIV testing programs do not specifically target older adults [16, 21, 22].

Line 110-11: 20,000/120,000 does not equal 18.3%. Please check actual numbers and correct.

According to the UNAIDS, it was estimated that among the 120,000 PLWH in Togo in 2019, 20,000 (16.6%) were aged ≥50 years [2].

Line 115: States the study was carried out in Lome, but in response to reviewer 1 it is stated the study was carried out in both Lome and other health regions in Togo. Please clarify, and if only hospitals in Lome were involved amend the title as suggested by Reviewer 1.

**Full title: HIV testing uptake and prevalence among hospitalized older adults in Togo: a cross-sectional study**

Line 181: See comment above – it is not clear how a point prevalence has a 95% CI?

**Authors: Please kindly refer to the response above.**

Line 206: p=<0.001 for both factors? Table 1 indicates other significant differences between men and women here eg monthly income, health insurance. Please mention these important differences in the text.

Women represented 43.9% of the sample and they were more likely to be widowed (p<0.001) and have no formal or primary level education (p<0.001).

Men were more likely to have health insurance (p<0.001) and have higher monthly income (p<0.001) compared with women.

Line 211: Please define the Mood test in Methods.

Comparisons of medians were performed using Mood test and proportions were compared using Chi-square test or Fisher’s exact test.

Line 223-4: Perhaps flip this result and state that 23 (57.5%) were newly diagnosed as a result of this testing.

Among HIV seropositive older adults, 23 (57.5%) were newly diagnosed as a result of this testing.

Table 1 and 2: Please correct age brackets to either 50-59 and ≥60 OR 50-60 and >60 (ie not 50-60 and ≥60).

*Discussion:
The additional studies relevant to Africa which have been included in the revised Discussion are of interest to gain context of the data generated in the present study. However, as written this revised Discussion lacks structure and logical flow and is far too long. It would be of use to briefly mention the data pertaining to HIV prevalence/testing in older people in Africa and compare with the current findings, then discuss possible reasons and suggested interventions. Some specific suggestions include (but are not limited to):*

Line 250-1: Where is the result indicating testing was not offered? State or remove sentence.

**Authors: We thank the editor for this remark. This statement was based on the results presented in figure 1. We added this result in the “result section” and stated it in the “discussion section”.**

Among the 619 older adults who were included in the study, 159 (25.7%) had never been tested for HIV and only 271 (43,7%) were tested during hospitalization, prior to our survey.

Line 274-76: Rephrase to say HIV testing campaigns targeting older people may be required.

Thus, HIV testing campaigns targeting older adults are needed to reach this group of populations.

Line 294 (and also line 337): The statement that there is limited data on HIV prevalence in older adults is inconsistent with the studies cited after, and in the Introduction, which detail a number of studies. Please rephrase.

Some studies have reported data on HIV prevalence in Africa among older adults aged ≥50 years.

This section (from lines 294 to 342) needs to be significantly shortened; group similar studies from different countries together, only include data relevant to older populations, critically compare and contrast results from this study with those previously reported and avoid long lists of prevalence data with minimal interpretation.

**Authors**: **We thank the editor for this comment. We have shortened this section of the discussion in the revised manuscript.**

Lines 338 and 342 – these statements seem to contradict?

**Authors**: **We have removed the statement in line 342.**
