# Supplementary material for: HIV testing uptake and prevalence among hospitalized older adults in Togo: A cross-sectional study
Source: PLoS One. 2021 Feb 2;16(2):e0246151. doi: 10.1371/journal.pone.0246151 (PMC7853528; doi:10.1371/journal.pone.0246151)
Supplement: Attachment — Submitted filename: Responses to the Editor.docx [file pone.0246151.s004.docx]

**Responses to the Editor**

*Keywords: “HIV uptake” should probably be “HIV testing” or “HIV testing uptake”. “Associated factors” is unlikely to be a useful search term – please remove/replace*

**Authors: We thank the Editor for these comments. We have modified keywords in the revised manuscript.**

**Keywords:** Ageing; HIV testing uptake; HIV prevalence; Togo.

*Line 53: Remove “infected” from this sentence.*

**Authors: We have removed the term ‘infected’ in the revised manuscript.**

The majority of older adults in this study accepted testing for HIV and almost three in five HIV positive older adults were newly diagnosed with HIV as a result of this testing.

*Line 109 – Should be 16.7%*

**Authors: We thank the Editor for this comment. We have corrected accordingly in the revised manuscript.**

According to the UNAIDS, it was estimated that among the 120,000 PLWH in Togo in 2019, 20,000 (16.7%) were aged ≥50 years [2].

*Line 128 – Please provide a reference for the SHOT study if published.*

**Authors: A more detailed description of the SHOT study has been previously published in PloS One. We provided the reference in the revised manuscript.**

25. Gbeasor-Komlanvi FA, Tchankoni MK, Bakoubayi AW, Lokossou MY, Sadio A, Zida-Compaore WIC, et al. Predictors of three-month mortality among hospitalized older adults in Togo. BMC Geriatr. 2020 Nov 26;20(1):507. doi: 10.1186/s12877-020-01907-y.

*Statistical analysis section (lines 177-): Please briefly explain in this section why Mood’s median test (which has a lower power than eg Mann-Whitney) was used. Please also explain why some categorical variables in Table 1 were assessed with Chi squared test and some with Fisher’s test.*

**Authors: We thank the reviewer for these comments. We added an explanatory paragraph in the statistical section.**

*;*

Comparisons of medians were performed using Mood’s Median test as it is more resistant to outliers and is more appropriate when the distributions in the sub-populations being compared differ (one symmetrical and the other asymmetrical). Proportions were compared using Chi-squared test when sample size of all cells was greater than 5 or Fisher’s exact test when at least one cell was below 5 [28].

*Line 202: Refer to Table 1 in line 202 when results first mentioned*

**Authors: This comment has been taken into account in the revised manuscript.**

The median age of the 619 participants was 61 years, (IQR: 55–70) (Table 1).

*Line 223-4: Regarding the use of the CI for the point prevalence, I thank the authors for the explanation and references provided. For the reader’s benefit please briefly explain in the text how these values were calculated and cite an appropriate reference or previous publication where you have explained this.*

**Authors: This comment has been taken into account in the revised manuscript.**

HIV prevalence was estimated with corresponding 95% confidence interval (95%CI) based on estimation of confidence interval for a proportion [27].

*Please also remove the CI from the abstract (line 49).*

**Authors: This comment has been taken into account in the revised manuscript.**

Of those who tested, forty patients were HIV positive, yielding a prevalence of 7.1%.

*Line 229-30 – Replace male and female with males and females (plural)*

**Authors: This comment has been taken into account in the revised manuscript.**

There was no statistical difference according to sex (6.9% in males, 7.2% in females; p=0.954).

*Line 245-6 – Sentence is repeated in line below – please remove*

**Authors: We thank the Editor for this remark. The sentence has been removed in the revised manuscript.**

Line 249 “In our study, one in four hospitalized older adults (25.7%) 250 had never been tested for HIV.” Repeats line 242-3 above. Remove 1 instance.

**Authors: We thank the Editor for this remark. We removed the sentence in the revised manuscript.**

*Line 255 Add replace “, whether” with “or whether” to make sentence grammatically correct.*

*Line 265: Change “health care providers feel uncomfortable…” to “health care providers* ***may*** *feel uncomfortable…”*

*Line 268: Add “an” before “alternative” to make sentence grammatically correct.*

*Line 277 DHS defined in line 271 – use abbreviation*

*Line 298  - change to “males” (plural)*

**Authors: We thank the Editor for these remarks. We corrected the sentences accordingly.**

*Line 255 Add replace “, whether” with “or whether” to make sentence grammatically correct*

Experience of HIV testing in older adults may depend on where they tested (hospital or community setting) or whether they actively sought the testing or not [22].

*Line 265: Change “health care providers feel uncomfortable…” to “health care providers* ***may*** *feel uncomfortable…”*

Besides, health care providers may feel uncomfortable discussing sexual history, especially with older adults [30].

*Line 268: Add “an” before “alternative” to make sentence grammatically correct.*

Also, an alternative approach with HIV self-testing could be proposed [31].

*Line 277 DHS defined in line 271 – use abbreviation*

While comparing with HIV prevalence observed in the general population, another review of data from 40 DHS conducted in 27 SSA countries between 2003 and 2012 found that HIV prevalence among adults (45-59 years) was higher than in the general adult population, except in Democratic Republic of Congo, Ethiopia, Mozambique, Sierra Leone, and Swaziland [33].

*Line 298  - change to “males” (plural)*

*Line 298  - change to “males” (plural)*

Based on available data in South Africa, the prevalence of HIV was 10.4% in males aged 50-54 years, 6.2% in males aged 55-59 years, and 3.5% in males aged ≥60 years [24].

*Line 330 – replace “more” with “better”*

**Authors: We thank the Editor for this comment. We have corrected accordingly in the revised manuscript.**

Moreover, the relationship between HIV infection and sexual practices among older adults should be better documented especially in West Africa.
